# Promotion of plant growth by endophyte *Bacillus amyloliquefaciens* N3 through modulation of auxin translocation under nitrate-limited conditions

**DOI:** 10.3389/fpls.2026.1775125

**Published:** 2026-03-13

**Authors:** Huan Luo, Yong Hu, Hong Shen, Xiaohui Zhang, Nannan Li

**Affiliations:** 1 College of Resources and Environment, and Academy of Agricultural Sciences, Southwest University, Chongqing, China; 2 Key Laboratory of Low-carbon Green Agriculture in Southwestern China, Ministry of Agriculture and Rural Affairs, Southwest University, Chongqing, China; 3 Interdisciplinary Research Center for Agriculture Green Development in Yangtze River Basin, Southwest University, Chongqing, China; 4 Yazhouwan National Laboratory, Sanya, China

**Keywords:** auxin transportation, *Bacillus*, endophytic bacteria, lateral root, limited-nitrate conditions

## Abstract

Numerous rhizobacteria have been isolated and could be utilized in an environmentally friendly way to improve crop yield by promoting plant growth. However, the underling mechanism is not well understood. In this study, we demonstrated that *Bacillus amyloliquefaciens* N3 isolated from purple soil promoted the lateral root formation and plant growth under nitrate-limited conditions. *B. amyloliquefaciens* N3 increased the local auxin accumulation in the lateral root tip, which may be attributed to the upregulated expression of *PIN-FORMED* (*PIN*) genes. Auxin polar transportation inhibition by NPA neutralized the promotion effects on lateral root density. Genetic analysis with mutants *auxin response regulator 7* (*arf7*), *arf9*, and *nitrated transporter 1.1* (*nrt1.1*) revealed that the promotion of plant growth by *B. amyloliquefaciens* N3 depends on both auxin-responsive ARF-mediated transcriptional regulation and *NRT1.1* under limited nitrate conditions. Stable promotion effects of *B. amyloliquefaciens* N3 on crop plants such as *Brassica napus* and *Zea mays* were validated in soil cultivation conditions. In summary, our study revealed further insights of the interaction mechanism between endophytic bacteria and plants and provided valuable resources for biofertilizer development.

## 1 Introduction

Plants have a high degree of plasticity in shaping their root architecture to optimize nutrient absorption. Lateral roots are important to forage for nutrients due to their ability to increase the uptake area of a root system (Pélissier et al., 2021). The NITRATE TRANSPORTER1.1 (NRT1.1) that functions in nitrate sensing and auxin transport plays a complex role in blocking lateral root primordia development (Pélissier et al., 2021). Under low nitrate conditions, NRT1.1 acts as an auxin transporter and facilitates basipetal transport of auxin away from the lateral root tip. This prevents the local auxin accumulation necessary for growth, thereby inhibiting lateral root elongation (Krouk et al., 2010). Conversely, under high nitrate conditions, NRT1.1 protein is downregulated in lateral root primordia, allowing auxin to accumulate (Bouguyon et al., 2016). Despite the regulation by NRT1.1, auxin accumulation in lateral root primordia is also affected by PIN1-, PIN3-, and PIN7-mediated auxin transportation (Lewis et al., 2011; Marhavý et al., 2014; Wang et al., 2019). This localized auxin buildup accelerates the degradation of AUXIN/INDOLE-3-ACETIC ACID proteins, thereby derepressing AUXIN RESPONSE FACTOR (ARF) proteins, such as ARF7 and ARF19 function as transcriptional activators. Once derepressed, ARF7 and ARF19 initiate the transcriptional programs required for lateral root primordium development and subsequent emergence (Okushima et al., 2007; Zhang et al., 2023).

Bacteria that colonize the roots of plants are essential contributors of plant growth. The genus *Bacillus* is one of the predominant bacterial genera found in soil and have been identified as plant growth-promoting rhizobacteria that improve plant performance through various mechanisms (Saxena et al., 2020). The *Bacillus subtilis* strain GB03 promotes cell expansion in *Arabidopsis* through volatile organic compound-dependent regulation of auxin homeostasis (Zhang et al., 2007). Another strain *Bacillus methylotrophicus* M4–96 isolated from the maize (*Zea mays*) rhizosphere increases growth and auxin content in *Arabidopsis thaliana* (Pérez-Flores et al., 2017). The most recent study revealed that the volatile compounds of *Bacillus amyloliquefaciens* SQR9 induced lateral root formation by regulating auxin biosynthesis (Li et al., 2021). In addition, *Bacillus* spp. can also help plants to tolerate abiotic stress caused by environmental factors such as drought, salinity, heat, and cold (Tsotetsi et al., 2022). The mechanisms associated with enhancing stress tolerance involve triggering biological and physiological processes such as ROS detoxification mechanisms, osmoprotection, stomatal regulation, membrane stability, xylem hydraulic conductance, root zone water, and nutrient availability and metal chelation (Mahapatra et al., 2022). While these studies shed light on the mechanisms of *Bacillus*-mediated plant growth promotion, a crucial gap remains: evaluating its effectiveness under conditions of nutrient stress, especially the nitrate-limited conditions. In this study, we explored the effects of strain *Bacillus amyloliquefaciens* N3 on lateral root development by utilizing molecular genetics approaches and validated its effects in different crop plants in soil conditions. Our finding expanded the understanding of beneficial endophytic bacterial application potential in nitrate-limited environments.

## 2 Materials and methods

### 2.1 Plant material and growth conditions

The *Arabidopsis thaliana* materials included the wild-type accession *Col-0*, the mutants *nrt1.1* (SALK_097431), *arf7* (SALK_027626C), and *arf19* (SALK_009879C), and the transgenic lines *pDR5*:*GFP*, *pPIN1:PIN1:GFP*, and *pPIN7:PIN7:GFP*, which were kindly provided by Prof. Ruifu Zhang (Nanjing Agricultural University). *Brassica napus* cultivar Zhongshuang 11 (ZS11) and *Zea mays* inbred line B73 were also used.

After 2 days of vernalization at 4°C in the dark, *Arabidopsis thaliana* seeds were immediately subjected to surface sterilization. Seeds were treated with 75% (v/v) ethanol for 1 min, followed by immersion in 10% (v/v) NaClO solution for 7 min, and then rinsed thoroughly five times with sterile distilled water. For *Brassica napus* ZS11 and *Zea mays* inbred line B73, seeds were sterilized using 75% (v/v) ethanol for 3 min, followed by 10% (v/v) NaClO solution for 5 min, and subsequently rinsed five times with sterile distilled water. The *Arabidopsis* and ZS11 seeds were grown in agar plates containing MS Base Salts (without nitrate) with addition of 1.9 or 0.19 g/L KNO_3_ in square petri plates (12×12 cm or 25×25 cm). Plates were then placed in the dark for 2 days to allow germination. Seeds with a similar germination state were chosen for inoculation experiments in plates. The 7-day-old ZS11 seedlings and germinated B73 seeds were used in the inoculation experiments in soil conditions.

For inoculation assays in plates, *B. amyloliquefaciens* N3 was cultured in Luria–Bertani (LB) liquid medium at 28°C with shaking at 180 rpm for approximately 16 h and then harvested by centrifugation at 5,000 × g for 10 min at room temperature, washed twice with sterile water, and resuspended in sterilized water to an optical density of OD_600_ = 0.1 ± 0.02. The inoculation medium was prepared by mixing the bacterial suspension with the indicated medium at a ratio of 1:10 (v/v). For mock treatment, sterile water prepared using the same procedure but without *B. amyloliquefaciens* N3 was mixed with the indicated medium at the same ratio. All subsequent growth conditions were identical between mock-treated and inoculated plants. Sterilized seeds with a comparable germination state were transferred to the inoculation medium and grown in a growth chamber at 22°C under a 16-h light/8-h dark photoperiod with a light intensity of approximately 200 μmol m^−2^ s^−1^.

For the inoculation experiments in soil conditions, plants were grown in the same batch of potting soil (total nitrogen, 1.36 g/kg^−1^; alkali-hydrolyzable nitrogen, 56 mg/kg^−1^, pH value, 6.42). Soil temperature was maintained according to the chamber settings, and soil texture was identical across all treatments. The 7-day-old ZS11 seedlings and germinated B73 seeds were transplanted into soil. Plants were grown in a growth chamber under the same conditions as described above. The bacterial inoculum was prepared as described above and resuspended with sterilized water to an optical density of OD_600_ = 0.1 ± 0.02. A 15-mL bacterial inoculum was inoculated into the root zone every week. Accordingly, the mock treatment was inoculated with 15 mL of sterilized water.

### 2.2 Phenotype evaluation and data analysis

For the phenotypes of plate inoculation experiments, seedlings inoculated for 12 days were scanned with an Epson Perfection V850 Pro scanner and further analyzed with ImageJ software for analysis of root phenotype. The total biomass of each seedling was measured on an analytical balance. For the phenotypes of soil inoculation experiments, at 14, 21, and 35 days after first inoculated treatment, seedlings were harvested for fresh weight evaluation.

### 2.3 Analysis of contents of indole-3-acetic acid and nitrate

Salkowski’s test was used to detect the presence of indole-3-acetic acid (IAA) or its precursors in the supernatant of bacterial culture as described before (Rahman et al., 2010; Gang et al., 2019). A salicylic acid-concentrated sulfuric acid colorimetry method (Qian et al., 2017) was used to determine the nitrate content in mock treatment and *B. amyloliquefaciens* N3-inoculated *Arabidopsis* plants.

### 2.4 N-1-naphthylphthalamic acid treatment and fluorescence microscopy

N-1-Naphthylphthalamic acid (NPA) was dissolved in dimethyl sulfoxide (DMSO) to make a 50-mM solution and supplied at different doses (1, 2, and 4 μM) to the plant growth medium. *Arabidopsis* plants grown in petri plates were placed in a growth chamber for 12 days to detect the phenotype of roots. For confocal microscopy, mock or *B. amyloliquefaciens* N3-inoculated DR5:GFP seedlings were mounted in distilled water on microscope slides. A Zeiss 780 laser-scanning microscope was used for fluorescence imaging of the *Arabidopsis* lateral roots at early stages of development as previously described (Malamy and Benfey, 1997). These stages were classified as the following: stage I, initiation of the lateral root primordium; stage II, periclinal division of the lateral root primordium into two distinct cell layers; stage III, further periclinal division of the outer layer, resulting in a three-layered primordium; stage IV, formation of a four-layered primordium; stages V–VII, expansion of the lateral root primordium through the parental cortex until it is poised to emerge from the primary root surface; and stage E, the emergence of lateral root. Chromophores were excited using a 488-nm argon laser, and fluorescence was detected at 500–550 nm. More than eight independent seedlings were analyzed for each genotype, and treatment representative images were selected for figure construction. In *pPIN1:PIN1:GFP* and *pPIN7:PIN7:GFP* transgenic lines, fluorescence signals were captured under identical microscope settings. Quantification was performed using ImageJ software. Measurements were performed on lateral root tip region, and the mean fluorescence intensity of each individual sample was calculated after subtraction of background fluorescence.

### 2.5 RNA extraction and quantitative real-time PCR analysis

The root materials collected after 12 days of co-cultivation were ground in liquid nitrogen, and the total RNA was extracted with three biological replicates. The cDNA synthesis was generated from total RNA using the PrimeScript™ RT reagent Kit (TaKaRa). Quantitative real-time (RT) PCR (q-PCR) was performed on a StepOnePlus™ Real-Time PCR System (Applied Biosystems) using a GoTaq qPCR Master Mix (Promega) and specific primers (**Supplementary Table 1**). *AtACTIN2* (AT3G18780) was used as the reference gene. The results were obtained from three biological replicates, and –ΔΔCt was taken for every sample as the relative expression levels.

### 2.6 Construction of mCherry-tagged *B. amyloliquefaciens* N3 strain and detection of its colonization in plants

The pUT-mini-Tn5-Kna-Tac-mCherry-SacB plasmid was transformed into *B. amyloliquefaciens* N3 by conjugation using a filter-mating method, which generates an mCherry-tagged strain *B. amyloliquefaciens* N3-mCherry. The 8-day-old *Arabidopsis* seedlings subjected to limited nitrate treatment were inoculated with *B. amyloliquefaciens* N3-mCherry strain in plates as described above. After 2 days, roots were carefully washed with sterile water three to four times and fixed with 1.5% agarose and then cut into 80 pieces with a vibrating microtome to obtain 80-μm sections. These sections were analyzed with Zeiss laser confocal microscope. At least 10 seedlings were treated, and three independent biological repeated experiments were carried out.

To detect *B. amyloliquefaciens* N3 colonization in the roots of *Brassica napus* ZS11, the *B. amyloliquefaciens* N3 strain was inoculated with 7-day-old ZS11 seedlings grown on plates, as described above. After 2 days of inoculation, root samples were collected and examined using scanning electron microscopy (JSM-5610, Phenom, Holland) at an accelerating voltage of 10 kV to observe bacterial colonization.

## 3 Results

### 3.1 *B*. *amyloliquefaciens* N3 promotes lateral root formation and plant growth under nitrate-limited conditions

**Figure 1:**
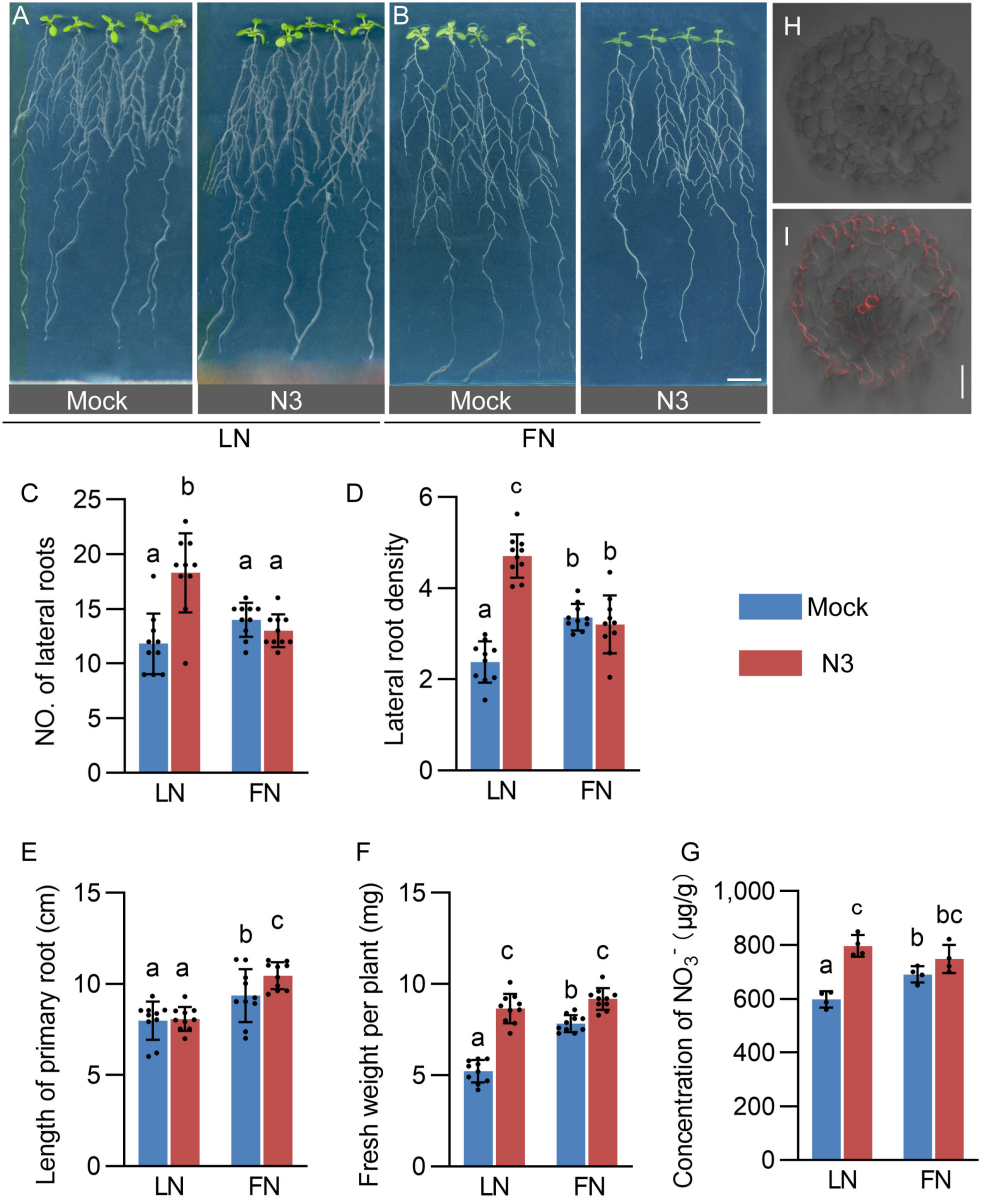
Promoted growth of *Arabidopsis* seedlings by *Bacillus amyloliquefaciens* N_3_. **(A, B)***Arabidopsis Col-0* seedlings co-cultivated with bacterial *B. amyloliquefaciens* N3 (OD_600_ = 0.1 ± 0.02) for 12 days in limited nitrate (LN) and full nitrate (FN) conditions. Scale bar, 1 cm. **(C-G)** Quantification of number of lateral root per plant **(C)**, lateral root density **(D)**, length of the primary root (PR) **(E)**, total biomass per plant **(F)**, and total NO_3_^-^ concentration of seedling after 12 days of co-cultivation **(G)**. Data are shown as mean ± standard deviation (SD), n = 15; different letters indicate the significant difference at *p* < 0.05, one-way ANOVA, Duncan’s test. **(H, I)** Fluorescence observation of the *B. amyloliquefaciens* N3 as the control **(H)** and mCherry-tagged *B. amyloliquefaciens* N3 **(I)** in the cross-cut section of *Arabidopsis* roots.

In our previous study, *B. amyloliquefaciens* N3 (hereafter referred to as N3) was isolated from purple soil, a specific type of soil found predominantly in regions like the Sichuan basin in China (Shen et al., 2018). N3 has been found to significantly promote the growth of Ginger (*Zingiber officinale* Roscoe) (Shen et al., 2018). In this study, we first confirmed the promotion effect on the growth of *Arabidopsis thaliana*. As expected, N3 increased the lateral root number, lateral root density, and total biomass but did not affect the primary root length per plant (**Figures 1A–F**). These effects were found more obvious under limited nitrate (LN, 0.19 g/L KNO_3_) than that in full-nitrate (FN, 1.9 g/L KNO_3_) conditions. In order to explore whether N3 can increase the absorption of nitrate by *Arabidopsis* and promote its growth and root development under LN treatment, the nitrate content in the plants was measured. The results showed that the nitrate content in the N3-inoculated *Arabidopsis* plants was significantly higher than that of the control group, but only under LN conditions (**Figure 1G**). The mCherry-tagged N3 line was constructed to trace the exact colonization region in the root. Strong signals were observed in the apoplastic spaces of epidermal and xylem tissues, weak fluorescence signals were also detected in the apoplast of the pericycle layer (**Figures 1H, I**). These results suggest that N3 is an endophytic bacterium.

**Figure 2:**
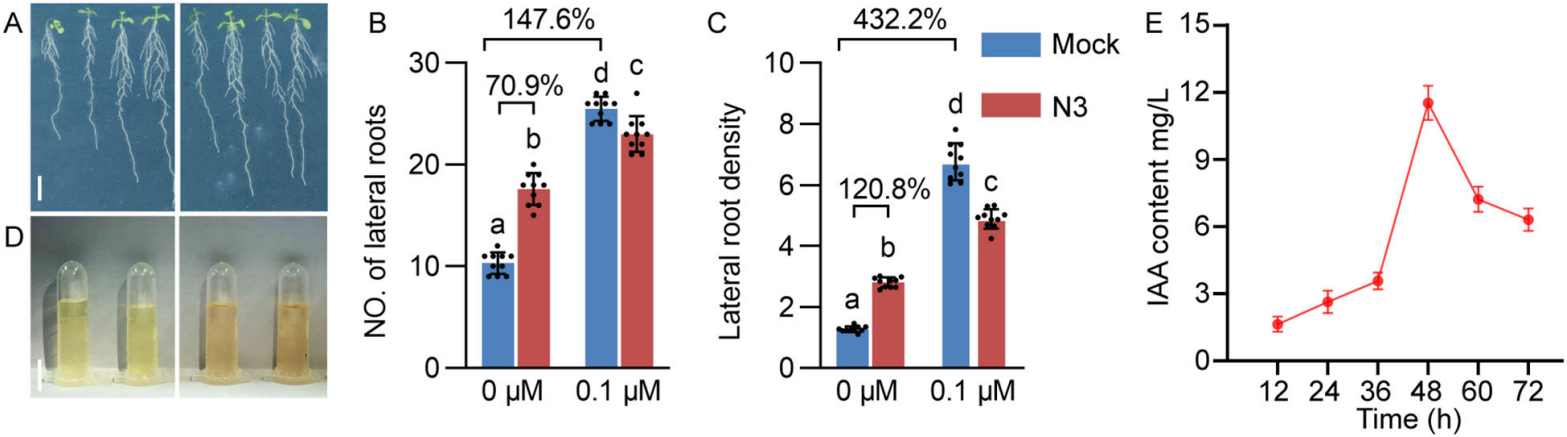
The effect of NAA treatment and *B. amyloliquefaciens* N3 inoculation on the root development of *Arabidopsis*. **(A)** The water-treated control (0 μM NAA, left) and 0.1 μM NAA (right) *Arabidopsis* plants. **(B, C)** Number of lateral roots **(B)** and lateral root density **(C)** of control and NAA-treated plants with or without inoculation of *B. amyloliquefaciens* N3; n ≥ 9. Percentages in the bar charts show the relative changes between the indicated bars; different letters indicate the significant difference at *p* < 0.05, one-way ANOVA, Duncan’s test. **(D)** Supernatant of control (left) and *B. amyloliquefaciens* N3 culture (right) treated with Salkowski’s reagent. **(E)** Quantitative analysis of IAA content in supernatant of *B. amyloliquefaciens* N3 culture; data are shown as mean ± SD, n=3. Scale bar, 1 cm in A and D.

The increase of lateral root number and density by N3 mimics the effects of exogenous auxin treatment, which motivated us to inspect the role of auxin in this process. We first compared the effects of N3 with the synthetic auxin, 1-naphthaleneacetic acid (NAA), on the growth of *Arabidopsis* roots. Treatment of N3 and NAA both increased the lateral root number and density, but more pronounced effects were observed in NAA treatment compared with N3 inoculation (**Figures 2A–C**). However, treatment of 0.1 μM NAA decreased the total biomass of *Arabidopsis* plants. Moreover, Salkowski’s test revealed that N3 exhibits IAA production potential and produces a maximum of IAA at 48 h post of culturing (**Figures 2D–E**). Taken together, these results showed that auxin may play an important role in shaping the root architecture by N3.

### 3.2 Upregulation of auxin-related genes after inoculation of N3

**Figure 3:**
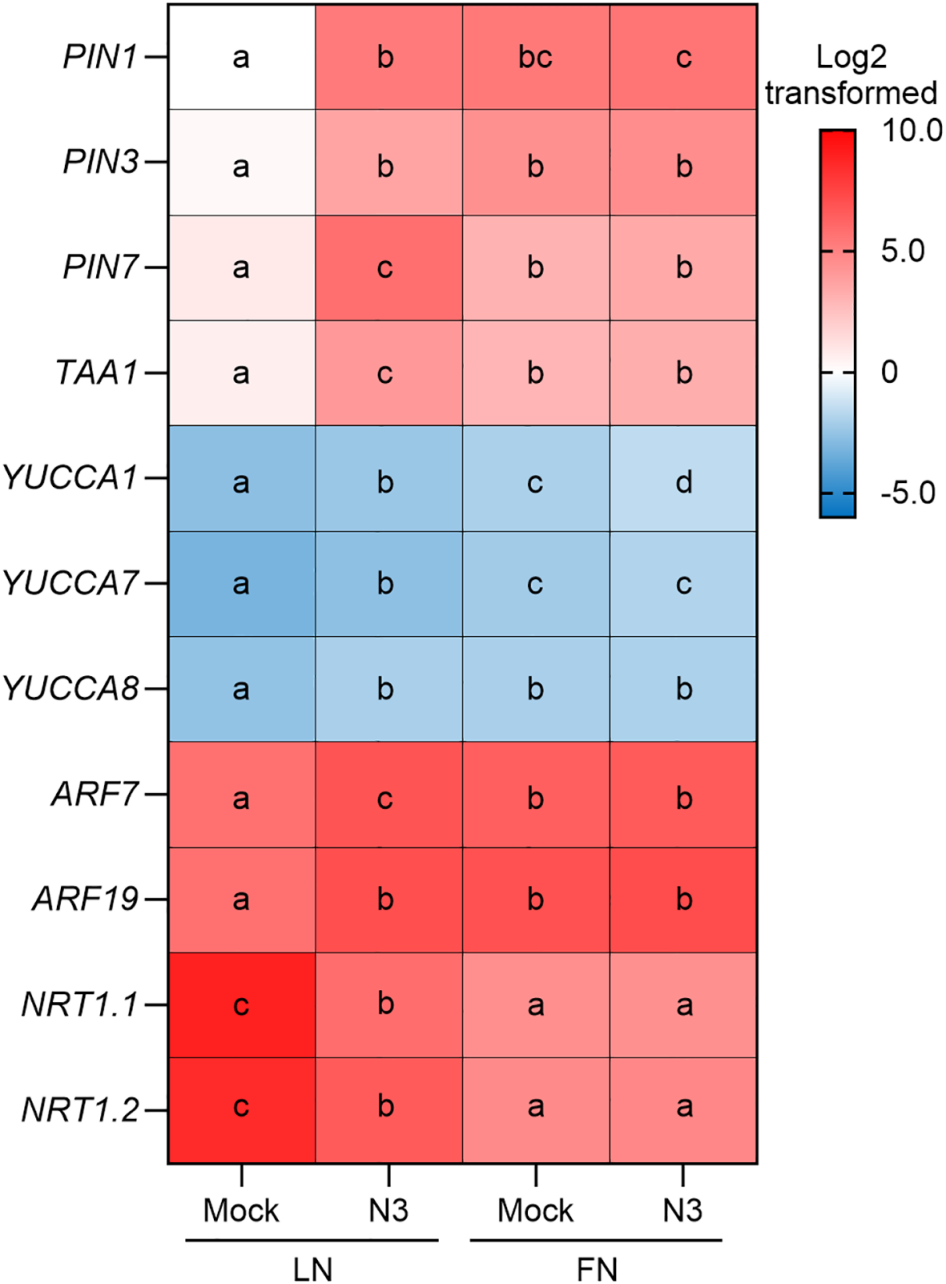
Expression analysis of gene involved in auxin-related genes and nitrate transporter genes. Expression levels of indicated genes in mock and *B. amyloliquefaciens* N3-inoculated 12-day-old plants under both LN and FN conditions. Data are shown as the Log2-transformed mean values of three replicates. Different letters indicate the significant difference at *p* < 0.05, one-way ANOVA, Duncan’s test.

To further dissect the involvement of auxin in the regulation of lateral root formation, we compared the expression levels of several auxin-related genes between N3-inoculated plants and control. Interestingly, under LN conditions, pronounced elevations of expression levels of *PIN1*, *PIN3*, and *PIN7* involving auxin transportation were detected when plants were inoculated with N3 (**Figure 3**). A similar pattern of expression changes was observed in the flavin monooxygenase genes, *YUCCA1*, *YUCCA7*, *YUCCA8*, and *TRYPTOPHAN AMINOTRANSFERASE OF ARABIDOPSIS 1* (*TAA1*) which regulate auxin biosynthesis, as well as in the two *Auxin Response Factor* (*ARF*) genes mediating the auxin-responsive regulation (**Figure 3**). These results suggest that N3 treatment transcriptionally altered the auxin biosynthesis, transport, and auxin-responsive ARF signaling in *Arabidopsis* plant.

### 3.3 N3-mediated lateral root development depends on auxin transport and ARF function

**Figure 4:**
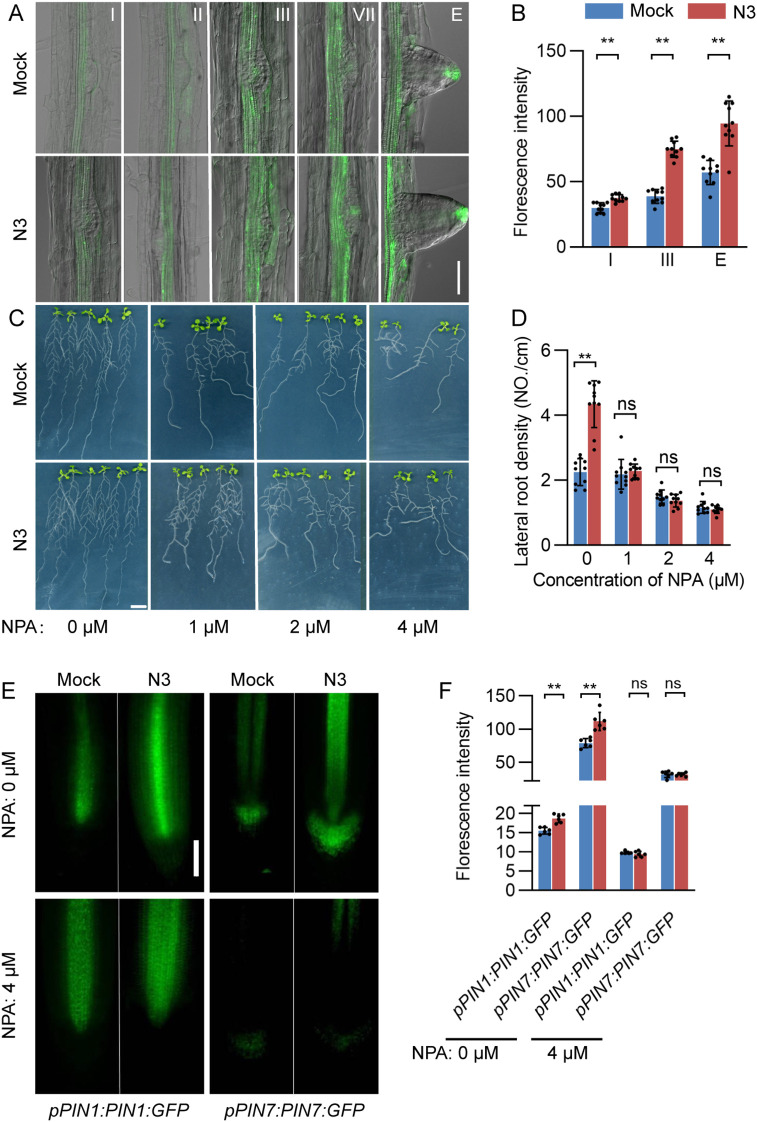
Auxin accumulation in the lateral root tip and its effects on lateral root development. **(A)** Expression patterns of *pDR5*:*GFP* during primordium development after 7 days of co-cultivation with *B. amyloliquefaciens* N3 detected with confocal laser scanning microscope; Scale bar, 50 μm. **(B)** Quantification of *pDR5*::*GFP* signal in the lateral root tips; n ≥ 9; **, *p* < 0.01, *t*-test. **(C)** Phenotype of NPA-treated plants with mock and *B. amyloliquefaciens* N3 treatment. **(D)** Lateral root density of NPA-treated plants with mock and *B. amyloliquefaciens* N3 treatment. Data are shown as mean ± SD, n ≥ 9, **, *p* < 0.01, ns, not significant, *t*-test. **(E, F)** GFP protein accumulation analysis of *pPIN1:PIN1:GFP* and *pPIN7:PIN7:GFP* signal **(E)** and quantification **(F)**; bar, 2 cm. Data are shown as mean ± SD, n = 6, **p < 0.01, ns, not significant, *t*-test.

The markedly increased expression of *PIN* genes inspires us to explore their roles in shaping lateral root architecture by N3. Local auxin distribution in the lateral root or its primordium was detected by using the *pDR5*:*GFP* lines. Significant enhancement of auxin signal was observed in the xylem and lateral root tip of N3-treated root compared to that of the mock plants at stages III to E (**Figures 4A, B**). A series of concentrations from 1 to 4 μM of NPA, a polar auxin transport inhibitor, were applied to block the auxin polar transportation in the N3-inoculated plants. In the NPA-free treatment, N3 treatment significantly increased the lateral root density (**Figures 4C, D**). The lateral root density decreased gradually with increases in NPA concentration. Most importantly, the NPA treatment totally abolished the promotion effects of N3 on the lateral root density regardless of the NPA concentrations. To further validate this, GFP-tagged PIN1 and PIN7 transgenic lines (*pPIN1:PIN1:GFP* and *pPIN7:PIN7:GFP*) were used to analyze the auxin transport protein accumulation upon NPA treatment (**Figures 4E, F**). As expected, PIN1 and PIN7 protein accumulation was enhanced by N3 inoculation. However, these effects were compromised when treated with 4 μM of NPA (**Figures 4E, F**). Thus, the promotion of lateral root development by N3 inoculation depends on the auxin polar transportation pathway.

**Figure 5:**
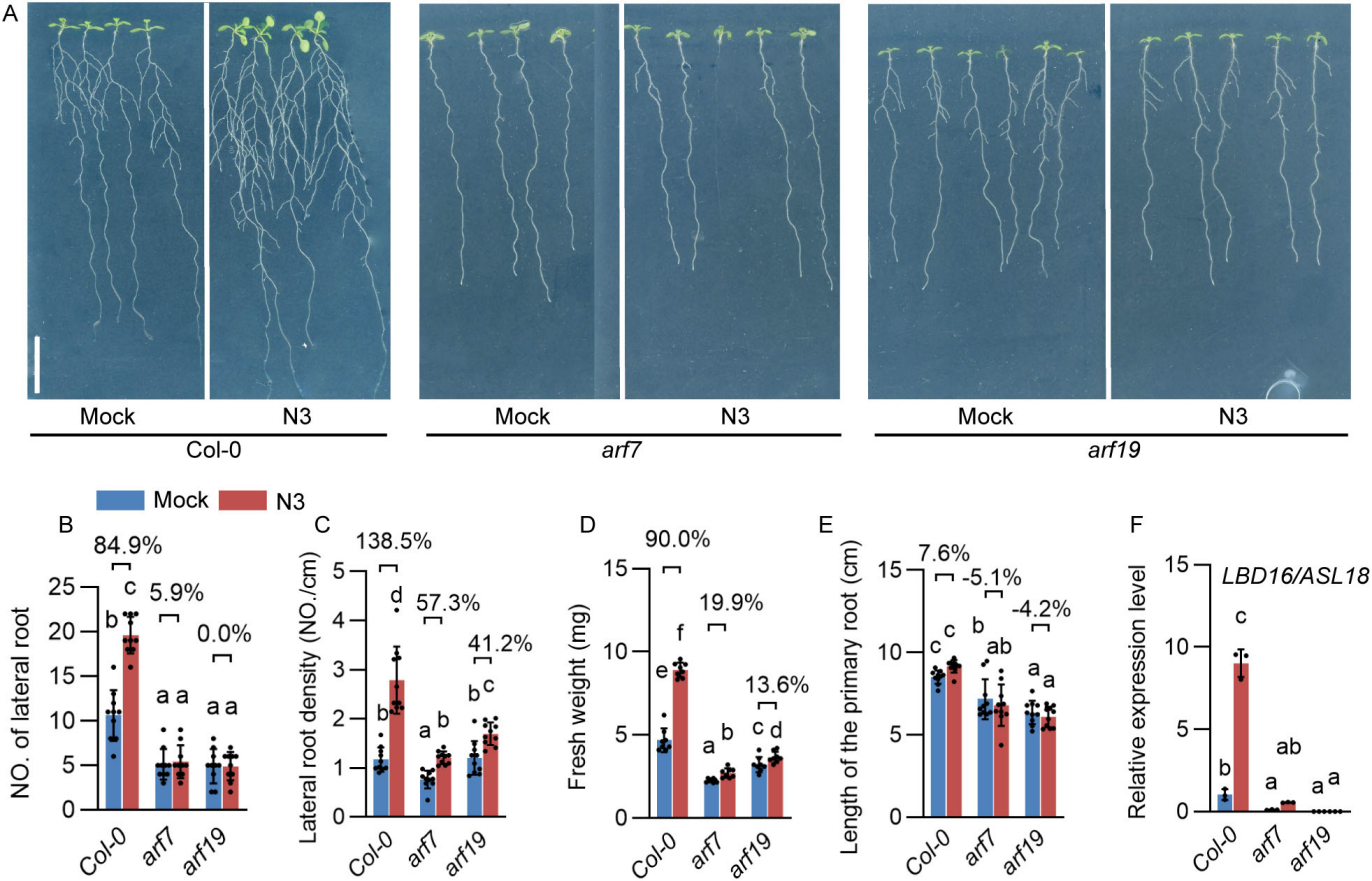
Effects of *B. amyloliquefaciens* N3 on growth and root development in *auxin response factor* (*arf*) mutants. **(A)** Phenotype of roots of *Col-0*, *arf7*, and *arf19* mutants with 12 days of mock or *B. amyloliquefaciens* N3 inoculation treatments, scale bar, 1 cm. **(B-E)** Number of lateral roots **(B)**, lateral root density **(C)**, fresh weight **(D)**, and length of primary root **(E)** after inoculation indicated in **(A)**; data are shown as mean ± SD, n ≥ 8. Percentages in the bar charts shows the relative changes between the indicated bars; different letters indicate the significant difference at *p* < 0.05, one-way ANOVA, Duncan’s test. **(F)** Expression analysis of *LOB DOMAIN-CONTAINING PROTEIN 16* (*LBD16*)/*ASYMMETRIC LEAVES2-LIKE 18* (*ASL18*) in Col-0, *arf7*, and *arf19* mutants. Data are shown as the mean ± SD of three replicates. Different letters indicate the significant difference at *p* < 0.05, one-way ANOVA, Duncan’s test.

Despite the upregulation of *PIN* genes, the expression levels of *ARF7* and *ARF19* were enhanced in the N3-inoculated plants. To analyze whether they mediated the interaction between N3 and plants, the phenotypes were compared between inoculated *Col-0* and *arf* mutants under LN and FN conditions (**Figure 5**, S1). In the *Col-0* plants, inoculation of N3 increased lateral root number by 84.9% compared to the control under LN conditions. However, this effect is totally neutralized in *arf7* or *arf9* backgrounds (**Figures 5A, B**). For the phenotype of lateral root density, lower percentages of increasement were observed in *arf7* (57.3%) and *arf19* (41.2%) than that in *Col-0* (138.5%) when inoculated with N3 (**Figure 5C**). A similar pattern was observed for fresh weight: N3 increased fresh weight by 90.0% in the *Col-0*, but only by 19.9% and 13.6% in *arf7* and *arf19*, respectively (**Figure 5D**). Moreover, we further performed expression analysis of *LOB DOMAIN-CONTAINING PROTEIN 16* (*LBD16*)/*ASYMMETRIC LEAVES2-LIKE 18* (*ASL18*) to validate the involvement of the *ARF*/*LDB* signaling pathway. Enhanced expression of *LDB16*/*ASL18* was detected in N3-inoculated *Col-0* plants. These effects were weakened in the *arf7* and abolished in *arf19* (**Figure 5F**). Taken together, our results demonstrated the promotion effect of N3 on the lateral root architecture depending on the auxin transport and auxin-responsive ARF signaling.

### 3.4 *NRT1.1* is crucial for enhancement of biomass by N3

**Figure 6:**
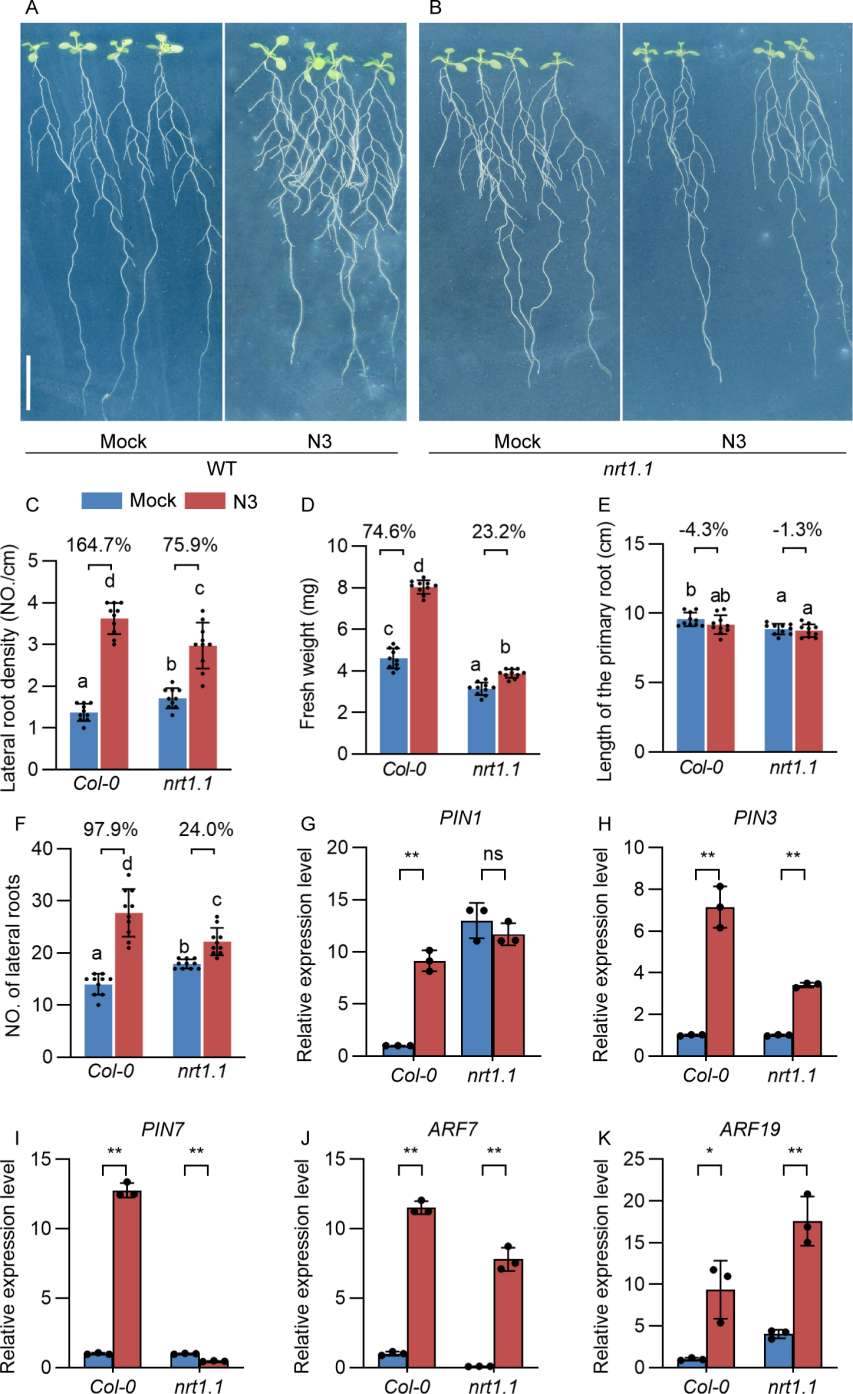
Effects of *B. amyloliquefaciens* N3 on growth and root development in *nitrate transporter 1.1* (*nrt1.1*) mutant. **(A, B)** Phenotype of roots of *Col-0* and *nrt1.1* mutant with 12 days of mock or *B. amyloliquefaciens* N3 inoculation treatments. **(C-F)** Lateral root density **(C)**, fresh weight **(D)**, length of the primary root **(E)** and the number of lateral roots **(F)**; data are shown as mean ± SD, n ≥ 9. Percentages in the bar charts shows the relative changes between the indicated bars; different letters indicate the significant difference at *p* < 0.05, one-way ANOVA, Duncan’s test, n ≥ 9. **(G-K)** Expression levels in *Col-0* and *nrt1.1* mutant under mock or *B. amyloliquefaciens* N3 inoculation treatments. Data are shown as the mean ± SD of three replicates. *p < 0.05; **p < 0.01, *t*-test.

The transcript level analysis also showed that several *nitrate transporter* genes were downregulated by N3 treatment under LN conditions (**Figure 3**). Moreover, *NRT1.1* was a key gene that is responsible for the root development at LN conditions (Krouk et al., 2010; Pélissier et al., 2021). To analyze its role in the N3-mediated plant growth promotion, we used the *nrt1.1* mutant in the inoculation experiment. Interestingly, the promotion of lateral root density was less effective in the *nrt1.1* mutant (75.9%) compared to that of *Col-0* (164.7%). Moreover, less effective promotion effects on fresh weight were observed in *nrt1.1* (23.2%) mutant compared with that in *Col-0* (74.6%) (**Figures 6A–F**, **Supplementary Figure 2**). These results indicated the significant but partial role of *NRT1.1* in the regulation of fresh weight during N3 inoculation. Because our results mentioned above demonstrated that N3-mediated regulation of lateral root formation totally relied on the auxin transport and auxin-responsive ARF-mediated pathways, we speculated that *NRT1.1* may act upstream of the auxin-related genes. To validate this hypothesis, we compared the expression of *PIN* and *ARF* genes between the inoculated and control groups in different backgrounds. As expected, the expression differences of *PIN1*, *PIN3*, and *PIN7* between N3-inoculated and mock plants were relatively smaller in *nrt1.1* than that in *Col-0* (**Figures 6G–K**). More specifically, expression levels of *PIN1* in *nrt1.1* showed no difference between mock and inoculated plants, which were distinct from the significant differences found in *Col-0* plants. Therefore, the influence on auxin transportation by N3 is partially dependent on *NRT1.1*.

### 3.5 N3 effectively promotes the growth of rapeseed and corn

**Figure 7:**
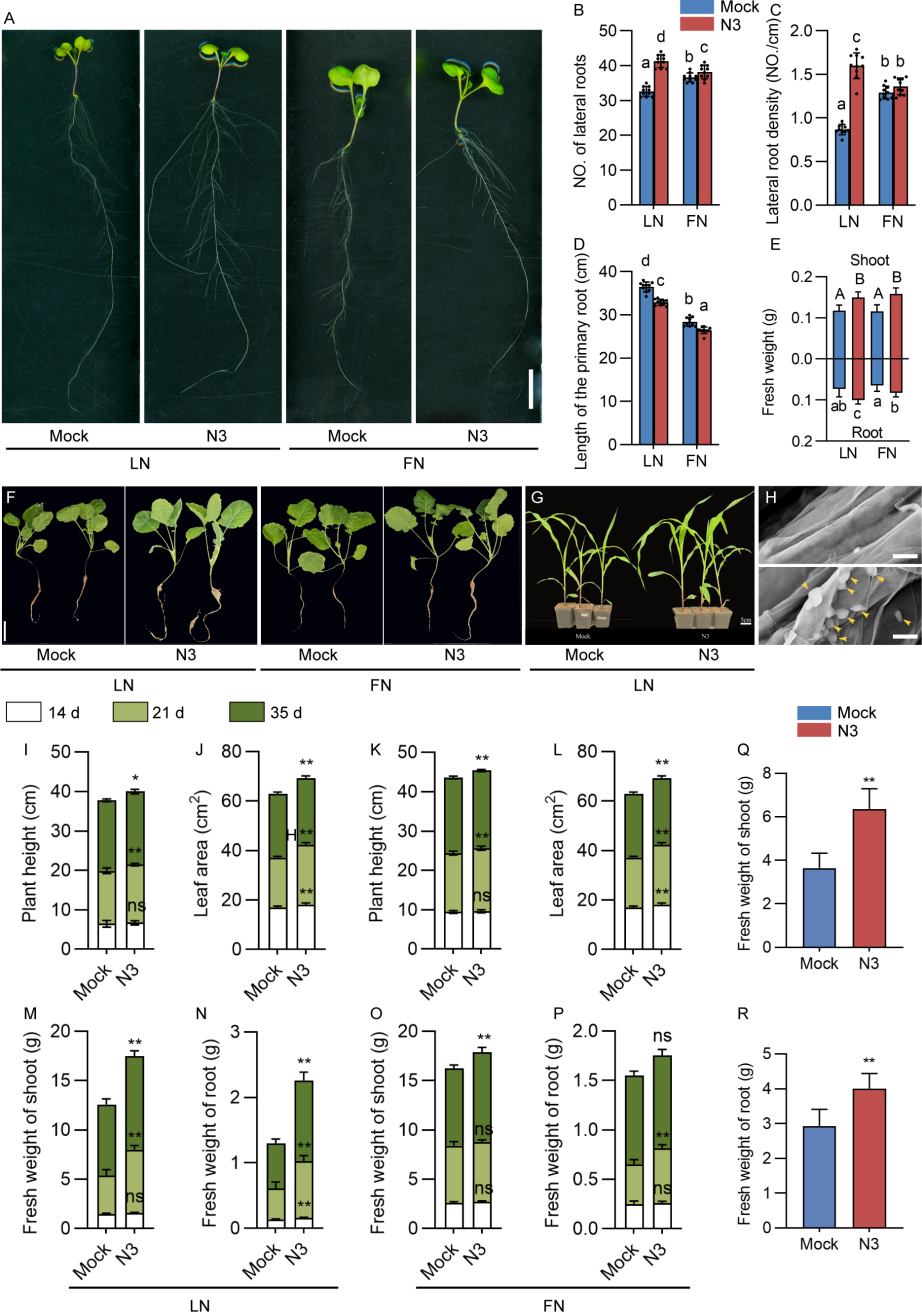
The promotion effect of *B. amyloliquefaciens* N3 on crop plants. **(A)** Promotion effect of *B. amyloliquefaciens* N3 on ZS11 under LN or FN conditions in the plate experiment. **(B-E)** Number of lateral root **(B)**, lateral root density **(C)**, length of primary root **(D)**, and fresh weight **(E)**; data are shown as mean ± SD, n ≥ 9. Different letters indicate the significant difference at *p* < 0.05, one-way ANOVA, Duncan’s test, n ≥ 9. **(F)** Seedling phenotypes of soil-grown ZS11 under LN or FN conditions with mock or *B. amyloliquefaciens* N3 inoculation; bar, 5 cm. **(G)** Phenotype of soil-grown *Zay maize* with mock or *B. amyloliquefaciens* N3 treatments under LN conditions; bar, 5 cm. **(H)** Colonization analysis of *B. amyloliquefaciens* N3 in root of *Brassica napus* ZS11 by using scanning electron microscope. Bar, 5 μm. Yellow arrows indicate the *B. amyloliquefaciens* N3 bacteria. **(I-P)** Plant height **(I, K)**, leaf area **(J, L)**, and fresh weight of shoot **(M, O)** and root **(N, P)** with mock or *B. amyloliquefaciens* N3 treatments under LN or FN conditions. Data are shown as the mean ± SD, n ≥ 9; *p < 0.05; **p < 0.01, ns, not significant, *t*-test. **(Q, R)** Fresh weight of corn shoot **(Q)** and root **(R)** with mock or *B. amyloliquefaciens* N3 treatments under LN conditions. Data are shown as the mean ± SD, n = 10; **, *p* < 0.01, *t*-test.

To evaluate the promotion effect of N3 on the crop plants, inoculation experiments were performed on dicot crop rapeseed and monocot crop corn (**Figures 7A–R**). In the plate experiments, N3 treatment significantly increased the lateral root number and density under LN conditions (**Figures 7A–E**). Significant increases of fresh weight of root and shoot were detected in N3-inoculated plants under both LN and FN conditions compared to the mock. The effects of N3 in the soil cultivation conditions were also evaluated (**Figures 7F, G**). Colonization of N3 in root of rapeseed was also validated with scanning electron microscopy (**Figure 7H**). For rapeseed, increases in plant height, leaf area, and fresh weight of shoot and root were detected after different days of inoculation (**Figures 7I–P**). These promotion effects were more obvious under LN compared with FN conditions, especially for the fresh weight traits. For the monocot corn, both shoot and root fresh weights were significantly increased by inoculation of N3 (**Figures 7G, Q, R**). These results suggested the N3 bacteria show consistent and stable promotion effects on different crop plants under soil conditions.

## 4 Discussion

In the model plant *Arabidopsis thaliana*, several genera of beneficial bacteria such as *Bacillus*, *Pseudomonas*, and *Phyllobacteria* strains can change the root system through different mechanisms including affecting plant hormone homeostasis or signal transduction (Kudoyarova et al., 2019; Dahmani et al., 2020; Jiménez-Vázquez et al., 2020). However, under the limited-nitrate conditions, the mechanism of promoting lateral root growth by beneficial bacteria is less investigated. In this study, N3 significantly increased the number of lateral roots, lateral root density, and nitrate content in *Arabidopsis* under nitrate-limited conditions. These promotion effects were very weak under FN conditions (**Figure 1**, **Supplementary Figure 3**). Our previous study has shown that N3 has N_2_-fixing activity (Shen et al., 2018). Thus, to understand the mechanisms behind plant promotion, it is important to determine whether it is caused by changes in auxin transport pathways or by enhanced N_2_ fixation. This study showed that the inoculation of N3 suppressed the expression of *NRT1.1* (**Figure 3**) and promoted the lateral root development (**Figures 1C, D**). This is different from the effects of direct application of external nitrate, which induces the expression of nitrate transporter genes and elicits lateral root elongation (Mantelin and Touraine, 2004). Therefore, changes in auxin transportation by N3 are more likely be the reason for enhancement of the root system which increased nitrate uptake (**Figure 1G**) and enhanced biomass accumulation (**Figure 1F**, **Figures 7E, O–R**).

**Figure 8:**
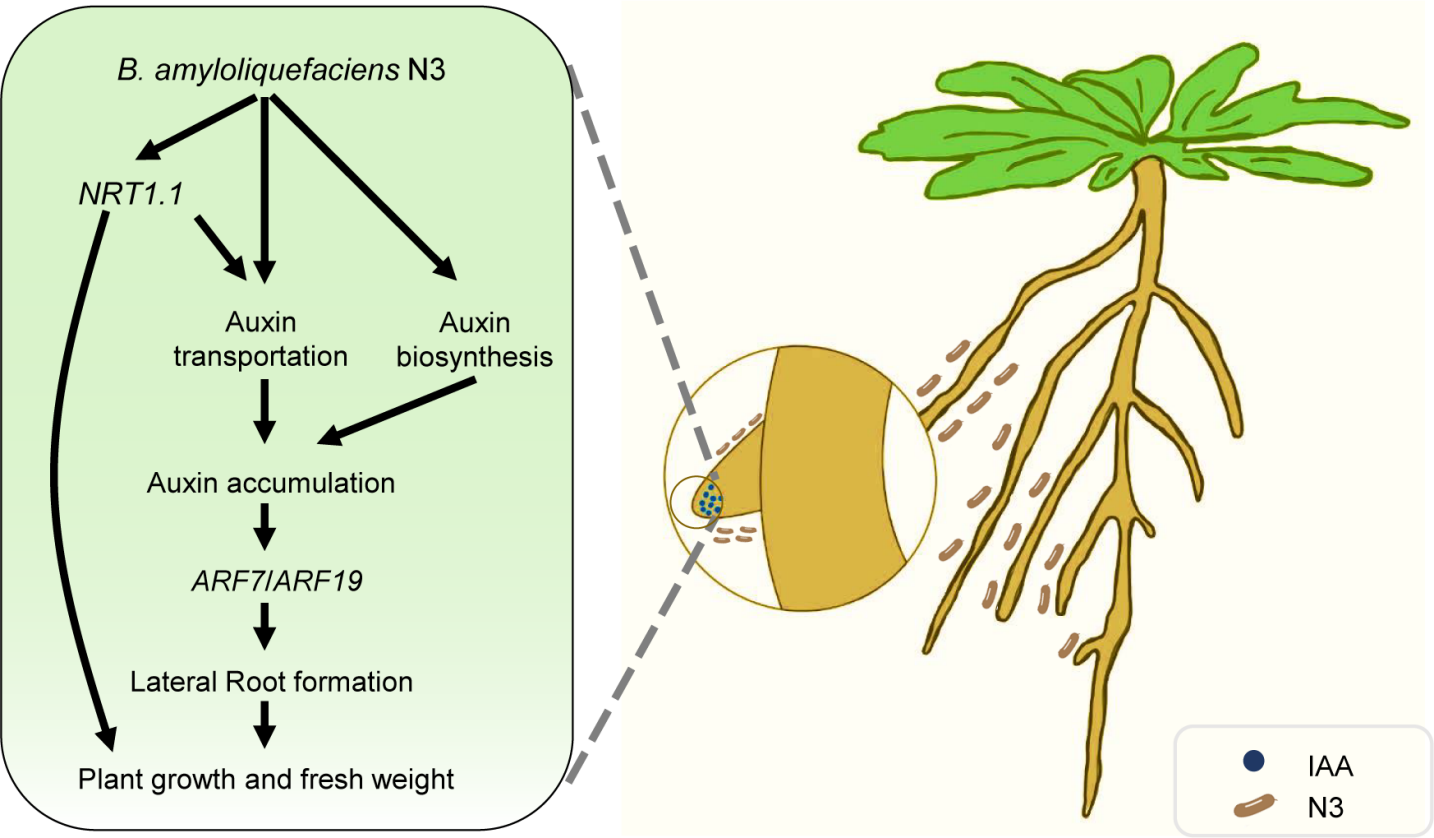
Model for *B. amyloliquefaciens* N3 promoting the development of lateral roots and plant growth. Under LN conditions, *B. amyloliquefaciens* N3 promoted the accumulation of auxin in the lateral root tips through affecting the auxin transportation mediated by PIN-FORMED and NRT1.1 proteins. *B. amyloliquefaciens* N3 also enhances the auxin biosynthesis by elevation of expression levels of *YUCCA* genes. The accumulated auxin enhanced the development of lateral roots through the *ARF7* and *ARF19* genes, which may promote the nutrient availability and plant growth.

*NRT1.1* was reported as a repressor of lateral root growth at low nitrate availability by promoting basipetal auxin transport (Krouk et al., 2010). In the present study, *NRT1.1* expression was transcriptionally suppressed upon N3 inoculation (**Figure 3**), and mutation of *nrt1.1* partially attenuated the N3-induced increase in lateral root density and fresh weight (**Figures 6C, D**). These results suggest that suppression of *NRT1.1* contributes, at least in part, to the promotive effect of N3 on lateral root development (**Figure 8**). In addition to *NRT1.1*, we observed enhanced accumulation of PIN1 and PIN7 proteins following N3 treatment (**Figures 4E, F**), indicating that PIN-mediated auxin transport is also strengthened. Thus, N3 appears to modulate auxin distribution through coordinated regulation of multiple transport components. It is noteworthy that mutation *arf7* or *arf19* did not completely compromise the promotion effects of N3 (**Figures 5C, D**), which suggested the existence of additional mechanisms that operate beyond ARF7/ARF19-dependent signaling. Given that NRT1.1 functions as an auxin transporter under limited nitrate conditions (Krouk et al., 2010), altered NRT1.1-dependent auxin transport may also contribute to the auxin accumulation in the lateral root tip. Furthermore, N3 inoculation significantly induced the expression of several auxin biosynthesis genes including *YUCCA1*, *YUCCA7*, *YUCCA8*, and *TAA1* (**Figure 3**). This is consistent with previous findings that constitutive overexpression of *YUCCA1* in the *yuc1D* mutant elevates IAA levels and enhances lateral root formation (Mashiguchi et al., 2011). Therefore, increased auxin biosynthesis triggered by N3 may represent another important mechanism underlying N3-induced lateral root initiation. Notably, the reduced promotive effect observed under exogenous NAA treatment may be explained by the elevated auxin levels provided by NAA, which could partially saturate auxin signaling and thereby attenuate the additional contribution of N3. Taken together, these findings indicate that N3 promotes lateral root development through coordinated modulation of auxin biosynthesis, auxin transport, and auxin-responsive ARF signaling, thereby reshaping local auxin homeostasis to favor lateral root formation (**Figure 8**).

Our results showed that the promotion functions of N3 was more pronounced under LN conditions (**Figures 1A–G**) compared with FN conditions (**Supplementary Figure 2**-**S3**). It correlated with the expression pattern that enhanced the expression of auxin-related genes, which was only observed under LN conditions (**Figure 3**). Considering that the promotion effect of bacteria was largely affected by the root exudation compounds (Jansson et al., 2023), we speculated that limited nitrate conditions may induce a specific compound secretion which may change the metabolism or growth state and enhance the effects of N3 in the root. On the contrary, metabolites seriated by the bacteria also influence the development and growth of host or nearby plants. The 6-pentyl-pyrone secreted by *Trichoderma atroviride* has been reported as a regulator of *Arabidopsis thaliana* root morphogenesis via auxin signaling and ETHYLENE INSENSITIVE 2 functioning (Garnica-Vergara et al., 2016). A similar mechanism may also exist in N3-mediated effects, which need further investigation in the future.

Although mCherry-tagged N3 was predominantly localized in the apoplastic spaces of epidermal and xylem cells, weak fluorescence signals were also detected in the apoplast of the pericycle layer (**Figures 1H, I**). As lateral roots originate from pericycle cells adjacent to the xylem pole (Pélissier et al., 2021), the presence of N3 in proximity to this cell layer suggests a potential spatial association between bacterial colonization and sites of lateral root initiation. While colonization was not strictly confined to the pericycle, this spatial proximity may facilitate local modulation of developmental signaling.

Under low nitrogen conditions, the growth-promoting effect of N3 exhibited a clear temporal pattern. Although no significant increase in plant height or shoot fresh weight was observed at 14 days after inoculation under LN conditions, leaf area and root fresh weight were already significantly enhanced at this early stage (**Figures 7J, L, N, P**). In contrast, significant increases in shoot biomass became evident at 21 and 35 days. This sequential response suggests that early stimulation of root growth may improve nutrient acquisition capacity, which subsequently supports aboveground biomass accumulation. Such a time-dependent enhancement is consistent with the notion that root system modulation represents an initial adaptive strategy under nitrogen limitation (Jia and von Wirén, 2020), ultimately leading to improved whole-plant performance. These findings further support the idea that N3-mediated regulation of root development plays a central role in promoting plant growth under LN conditions.

Although volatile compounds of *Bacillus amyloliquefaciens* SQR9 promote lateral root development (Li et al., 2021), our findings demonstrated that N3 is an endophyte, because it is detected in the apoplast and the vascular bundles (**Figures 1H, I**). N3 promotes nitrogen fixing and nitrate uptake into roots under LN conditions. Despite the growth promotion effects on *Arabidopsis*, our results have also revealed the consistent effects on both dicot crop *Brassica napus* and monocot crop *Zea mays* (**Figure 7**). Thus, the endophyte N3 may have great potential in application as microbial fertilizer.

In conclusion, our study demonstrates that N3 promotes lateral root development and enhances plant growth under low nitrogen conditions. Mechanistically, N3-mediated growth promotion depends on auxin transport and ARF function and is accompanied by transcriptional changes in auxin biosynthesis-related genes. Soil-based experiments further confirm that N3 improves plant performance in an agronomically relevant context. Together, these findings provide new insights into the interaction between beneficial rhizobacteria and auxin-regulated root development under nitrogen limitation.

## Data Availability

The original contributions presented in the study are included in the article/**Supplementary Material**. Further inquiries can be directed to the corresponding author.
