# Supplementary material for: Promotion of plant growth by endophyte Bacillus amyloliquefaciens N3 through modulation of auxin translocation under nitrate-limited conditions
Source: Front Plant Sci. 2026 Mar 13;17:1775125. doi: 10.3389/fpls.2026.1775125 (PMC13021573; doi:10.3389/fpls.2026.1775125)
Supplement: Supplementary file 1 [file DataSheet1.docx]

**Supplementary files (Figures and Tables)**


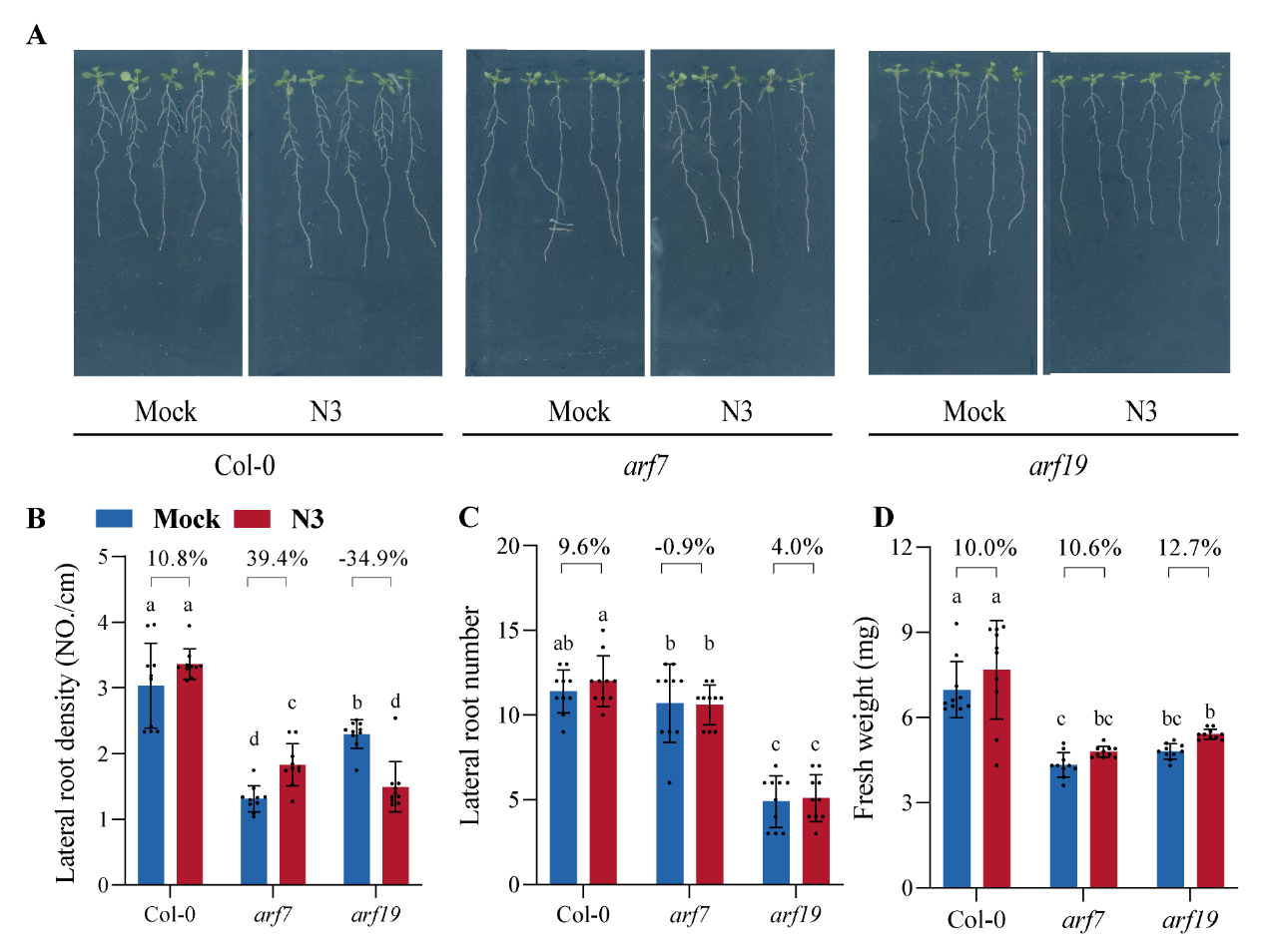


**Figure S1. Effects of *Bacillus amyloliquefaciens* N3 on the growth and root development in *auxin response factor* (*arf*) mutants under full nitrate (FN) conditions.**

(A) The phenotype of roots of *Col-0*, *arf7* and *arf19* mutants with 12 days of mock or *B. amyloliquefaciens* N3 inoculation treatments; (B-D) The lateral root density (B), the number of lateral roots (C), and fresh weight (D) after inoculation indicated in (A). Data are shown as mean ± SD, n ≥ 8; percentages in the bar charts shows the relative changes between the indicated bars; different letters indicate the significant difference at *p* < 0.05, one-way ANOVA, Duncan’s test.


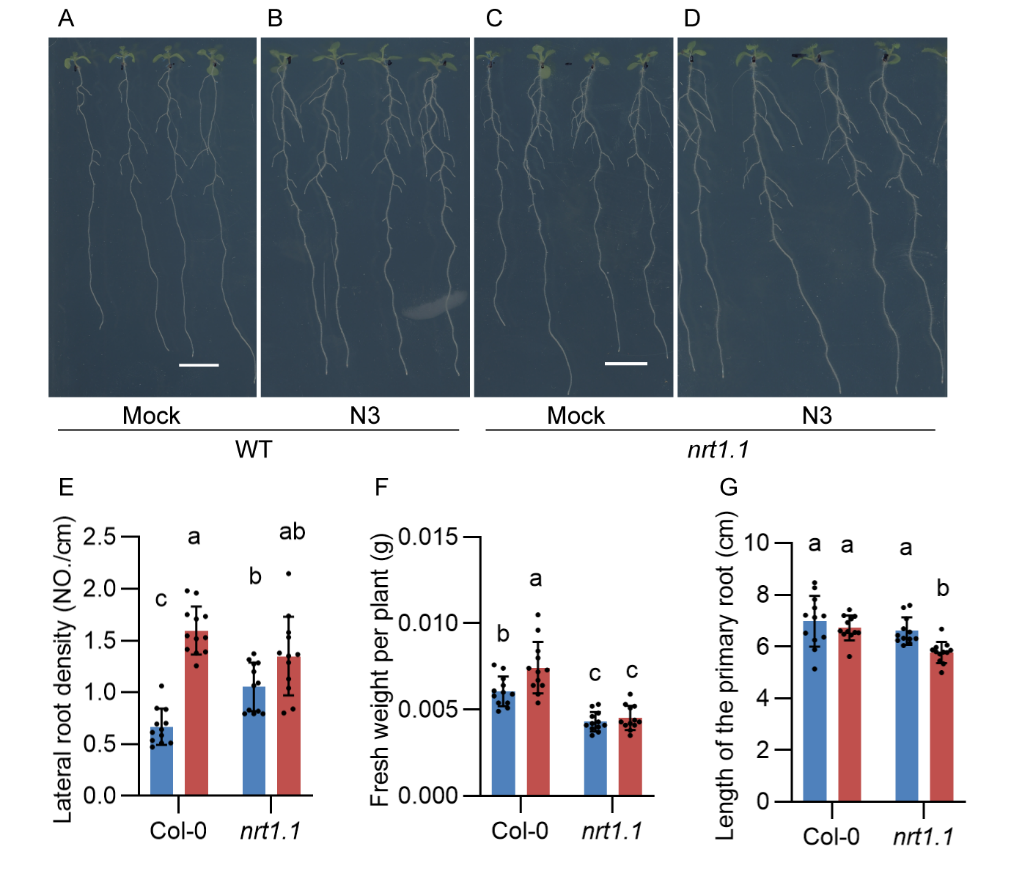


**Figure S2. Effects of *B. amyloliquefaciens* N3 on growth and root development in *nitrate transporter 1.1* (*nrt1.1*) mutant under FN conditions.**

(A-D) The phenotype of roots of *Col-0* and *nrt1.1* mutant with 10 days of mock or *B. amyloliquefaciens* N3 inoculation treatments; (E-G) Lateral root density (E), fresh weight (F), length of primary root (G); data are shown as mean ± SD; different letters indicate the significant difference at *p* < 0.05, one-way ANOVA, Duncan’s test, n ≥ 9.


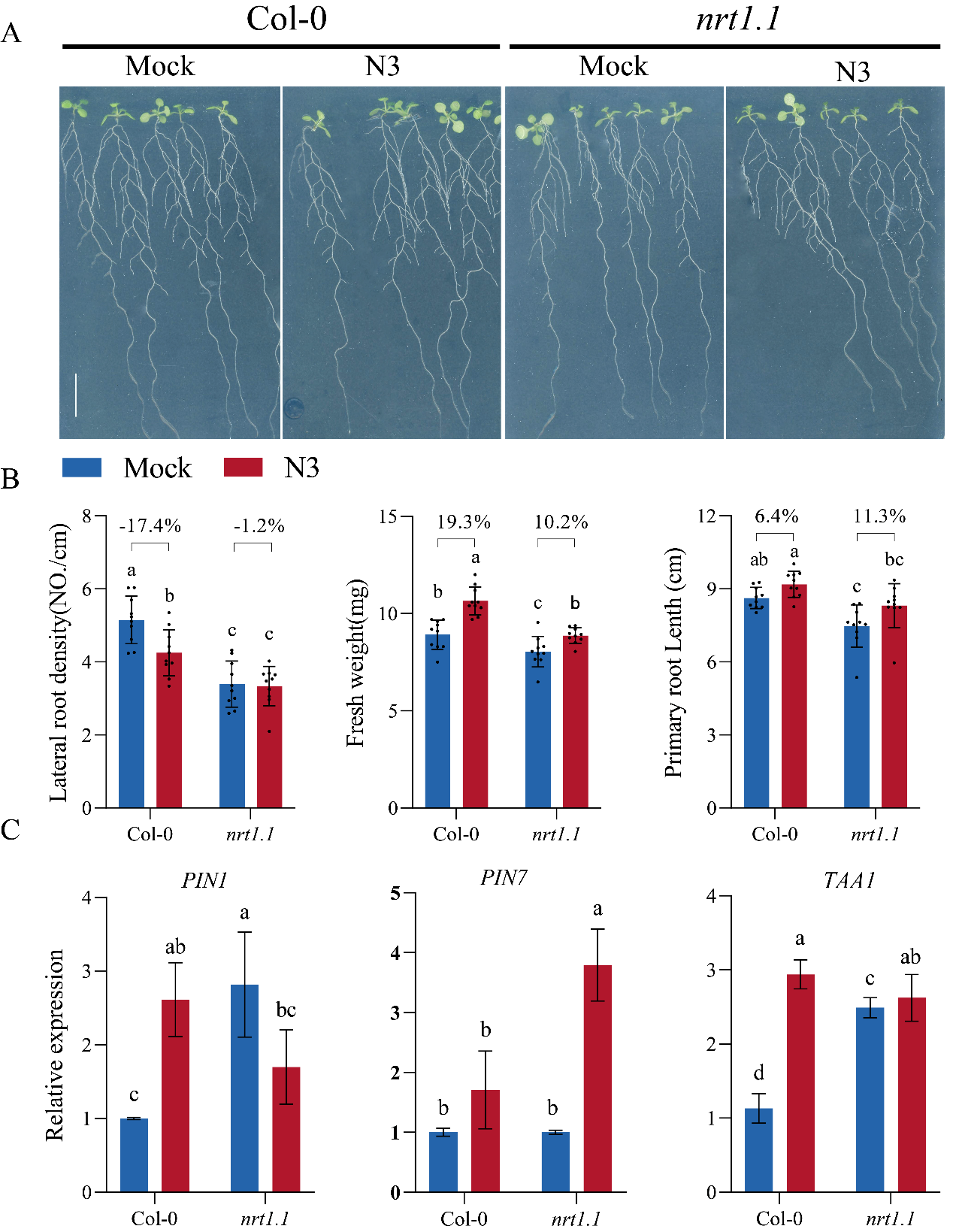


**Figure S3. Effects of *B. amyloliquefaciens* N3 on growth and root development in *nitrate transporter 1.1* (*nrt1.1*) mutant under FN conditions**

(A) The phenotype of roots of Col-0 and *nrt1.1* mutant with 12 days of mock or *B. amyloliquefaciens* N3 inoculation treatments; (B) The number of lateral roots, fresh weight, and length of primary root under FN conditions; data are shown as mean ± SD, n ≥ 9; percentages in the bar charts shows the relative changes between the indicated bars; different letters indicate the significant difference at *p* < 0.05, one-way ANOVA, Duncan’s test, n ≥ 9. (C) Expression levels in Col-0 and *nrt1.1* mutant under mock or *B. amyloliquefaciens* N3 inoculation treatments. Data are shown as the mean ± SD of three replicates; different letters indicate the significant difference at *p* < 0.05, one-way ANOVA, Duncan’s test.

**Table S1. Primers used in this study**

| Primername | Sequence(5’-3’) |
| --- | --- |
| BP-SALK | ATTTTGCCGATTTCGGAAC |
| nrt1.1-LP | GCAAGCGACTATCATCACTCC |
| nrt1.1-RP | GTTCTCCATGAGCTTCGTGAG |
| arf7-LP | ACCGAGGATTTGTGAAAACTG |
| arf7-RP | CAGCTAGATCGTTCGAAATGG |
| arf19-LP | AGCTGCAAGTATCCCAATGTG |
| arf19-RP | GCGAGCAAGTAAGTTCATTGC |
| AtActin2-fwd | TTTCCCGCTCTGCTGTTGT |
| AtActin2-rev | TGTGCCAATCTAVGAGGGTTT |
| AtPIN1-fwd | CGGTGGGAACAACATAAGCA |
| AtPIN1-rev | GGTGATGCCGAATAAACTGGA |
| AtPIN2-fwd | CCGTGGGGCTAAGCTTCTCATCT |
| AtPIN2-rev | AGCTTTCCGTCGTCTCCTATCTCC |
| AtPIN3-fwd | TCTTTGATTAGGTTCGGGTAACTC |
| AtPIN3-rev | GCTCATGTGAAACTGGAACAAG |
| AtPIN7-fwd | CGTGGCAGCAATGGCTATTGGATT |
| AtPIN7-rev | AAACACAAACGGCACGATCCCTTG |
| AtTAA1-fwd | TCCATTGGTGTGTCGAAGGA |
| AtTAA1-rev | AAACGCAGGAGAAGTGGAGA |
| AtARF7-fwd | CGAATTCGCTCCTTCATCAAATG |
| AtARF7-rev | CGGATCCTCAAGGACATATGTAGAAAG |
| AtARF19-fwd | CAGCAGCAAGGACAGATGAGTAAC |
| AtARF19-rev | GGAAGGTGAGGTTGAACAAGAAGG |
| AtYUCCA1-fwd | TCCGGTTCTCGATGTCGGAG |
| AtYUCCA1-rev | GGGTTATGGGAATCATGACGAC |
| AtYUCCA7-fwd | TGAGTACCTTGAGTCCTA |
| AtYUCCA7-rev | AACCTCTTGTCATATTTAGC |
| AtYUCCA8-fwd | AGACCCAAAGCGAATCAA |
| AtYUCCA8-rev | GCCACATCTTCCTCTGTT |
| AtNRT1.1-fwd | CTGCCACACACTGAACAATTCC |
| AtNRT1.1-rev | CCCGCTTCCTGATCCCTTAT |
